# Acalculous Cholecystitis in a Patient with Hepatocellular Carcinoma on Sorafenib

**DOI:** 10.5402/2011/201529

**Published:** 2010-11-23

**Authors:** Mariko Sanda, Hideyuki Tamai, Hisanobu Deguchi, Yoshiyuki Mori, Kosaku Moribata, Naoki Shingaki, Kazuki Ueda, Izumi Inoue, Takao Maekita, Mikitaka Iguchi, Kimihiko Yanaoka, Masashi Oka, Masao Ichinose

**Affiliations:** Second Department of Internal Medicine, Wakayama Medical University, 811-1 Kimiidera Wakayama City, Wakayama 641-0012, Japan

## Abstract

A 67-year-old woman with compensated cirrhosis type B associated with hepatocellular carcinoma was started on sorafenib for multiple pulmonary metastases. The patient developed right upper quadrant pain and high fever 4 weeks later. Imaging revealed marked enlargement of the gallbladder without calculi. Following percutaneous transhepatic gallbladder aspiration, her symptoms resolved, but the gallbladder remained enlarged. Laparoscopic cholecystectomy was performed. Arteriolar occlusion with intimal thickening in the muscular layer of the gallbladder was seen sporadically. The fact that this patient had no risk factors for acalculous cholecystitis suggested that the cholecystitis resulted from ischemia, implying a strong causal relationship with sorafenib.

## 1. Introduction

Sorafenib is a multikinase inhibitor that targets the cellular signal transduction pathways necessary for tumor cell proliferation and angiogenesis [1, 2]. Clinically, sorafenib is the first molecular targeted agent to inhibit tumor progression and prolong survival in hepatocellular carcinoma (HCC) [3, 4]. Sorafenib was approved in Japan in May 2009 for unresectable advanced HCC, and its use is expanding. We report herein a patient on sorafenib for pulmonary metastases of HCC with complicating acute acalculous cholecystitis who required cholecystectomy. To the best of our knowledge, this represents the first reported case of acalculous cholecystitis developing during sorafenib therapy, and is significant as a causal relationship with sorafenib was strongly suggested.

## 2. Case Report

**Figure 1:**
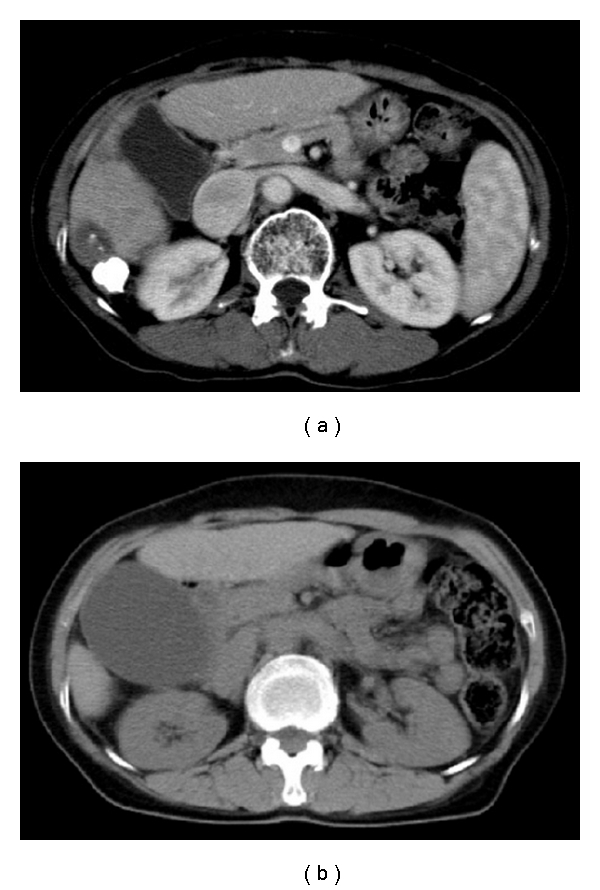
(a) Gallbladder swelling cannot be seen on baseline abdominal CT before sorafenib administration. (b) A highly tense and enlarged gallbladder can be seen on abdominal CT. There is no thickening of the gallbladder wall. The intrahepatic and common bile ducts are not dilated, and there are no calculi in the gallbladder or tumorous lesions in the neck of the gallbladder.

**Figure 2:**
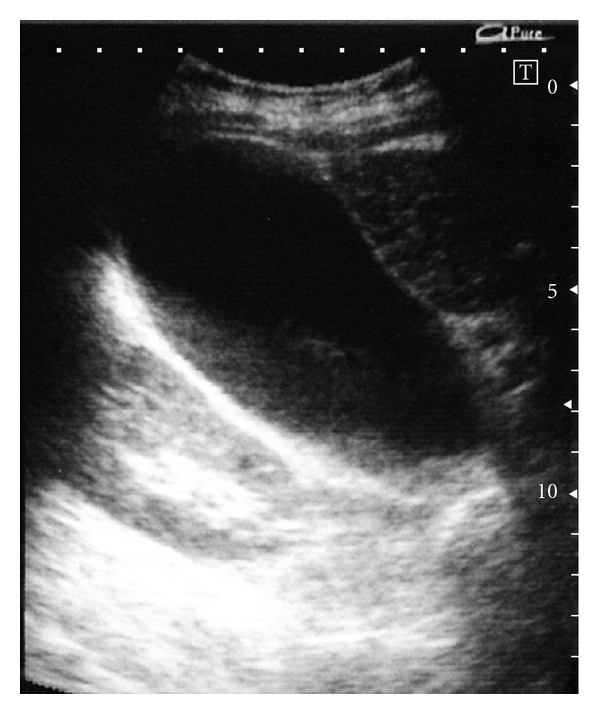
Clear thickening of the gallbladder wall cannot be seen on abdominal ultrasonography, but echoes from biliary debris can be seen inside a highly tense and enlarged gallbladder. Clear elevated lesions and calculi cannot be seen in the gallbladder.

**Figure 3:**
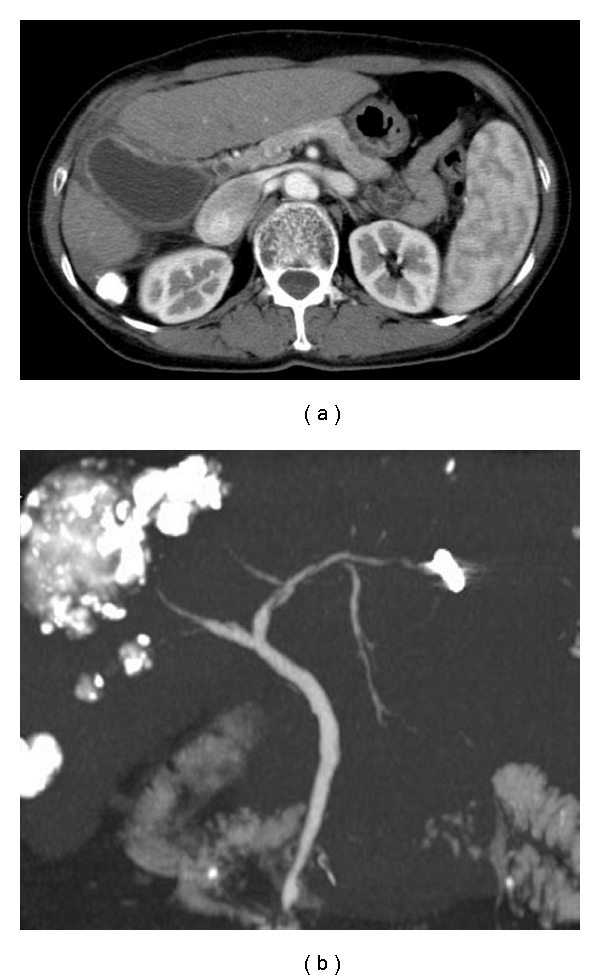
(a) Gallbladder swelling with wall thickness remained on abdominal CT 30 days after admission. (b) Gallblader and cystic duct were not visualized on three-dimensional spiral CT cholangiography.

**Figure 4:**
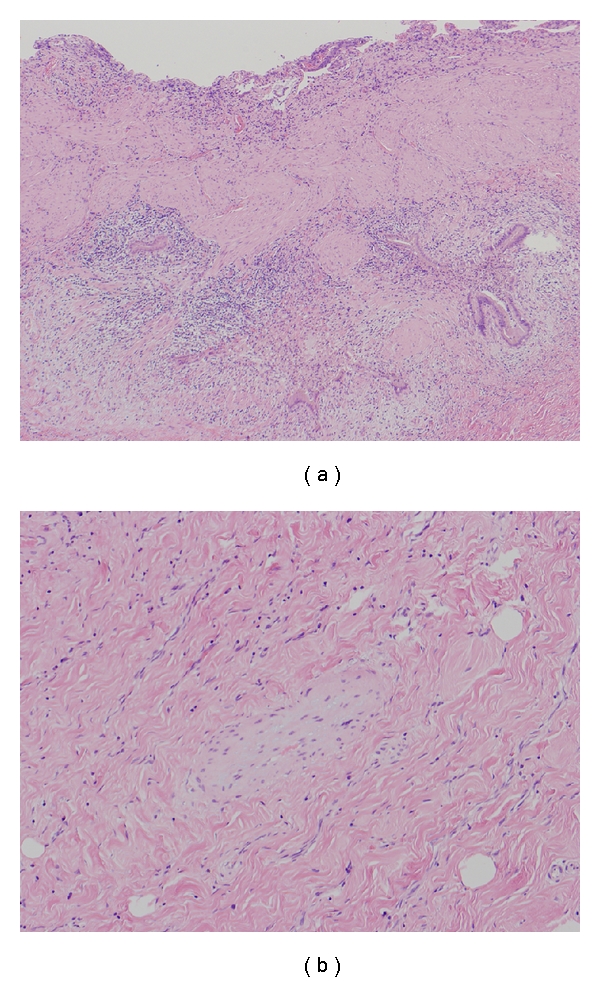
(a) Changes due to chronic cholecystitis can be seen: proliferation of fibromuscular tissue and formation of Rokitansky-Aschoff sinuses can be seen on the gallbladder wall. (b) Occluded arterioles with thickened vascular endothelium can be seen in the muscular layer of the gallbladder.

The patient was a 67-year-old woman with cirrhosis type B who was referred to our department by her local physician for two HCC lesions (86 mm in S8 and 23 mm in S6). Our Department of Surgery determined that the HCCs were unresectable due to poor hepatic functional reserve. Lipiodol transcatheter arterial chemoembolization (Lip-TACE) was performed with subsequent radiofrequency ablation (RFA). In addition, entecavir was started for the cirrhosis type B. There was recurrence of the multiple intrahepatic metastases 11 months later, for which Lip-TACE with RFA was performed. Thirty-one months later, there were innumerable pulmonary metastases bilaterally, and sorafenib, 800 mg daily, was started. When sorafenib therapy commenced, her Eastern Cooperative Oncology Group performance status (PS) was 0, her platelet count were 7.9 × 10^4^/mm^3^, and her Child-Pugh score was 5 points (Class A). She had no history of concurrent diabetes mellitus, hypertension, ischemic heart disease, or thromboembolism, nor did she have marked cytopenia or renal dysfunction that would have been of concern. She did not experience any adverse reactions after starting sorafenib, except for Grade 2 hypertension and Grade 1 hand-foot skin reaction, graded according to the Common Terminology Criteria for Adverse Events, Version 3.0. Four weeks after starting sorafenib, the patient developed right upper quadrant pain (RUQ) and high fever, for which she received emergency treatment in our department. Blood examination revealed an inflammatory reaction, with a white blood cell count of 6470/mm^3^, a neutrophil left shift of 83%, and a C-reactive protein level of 5.73 mg/dL. Although a tendency toward disseminated intravascular coagulation (DIC), with a marked decrease in platelet count (3.4 × 10^4^/mm^3^), prothrombin time INR of 1.29, and an increase in fibrin degradation products (15.8 *μ*g/dL) was suspected, a fibrinogen 629 mg/dL and DIC score was four. The following laboratory values showed no marked changes from pre-sorafenib levels: total bilirubin: 1.7 mg/dL; direct bilirubin: 0.2 mg/dL, albumin: 3.9 g/dL; aspartate aminotransferase: 53 IU/L; alanine aminotransferase: 30 IU/L; alkaline phosphatase: 164 IU/L; *γ*-glutamyl transpeptidase: 20 IU/L. Abdominal plain computed tomography revealed a markedly enlarged gallbladder (Figure 1), and abdominal ultrasound (Figure 2) revealed biliary debris without calculi. She was diagnosed with acute acalculous cholecystitis, and was admitted on an emergency basis. Sorafenib was discontinued, and sulbactam sodium, 1 g daily, was started. However, her symptoms did not improve and DIC score increased on day 2 after admission, percutaneous transhepatic gallbladder aspiration was performed. *Pseudomonas aeruginosa* and *Serratia marcescens* were identified in the bile. The cholecystitis resolved immediately postoperatively, but the gallbladder remained enlarged (Figure 3), and the RUQ discomfort persisted. Therefore, laparoscopic cholecystectomy was performed 45 days after admission. The gallbladder showed no calculi or neoplastic changes, but macroscopic adenomyomatosis was seen in the fundus. Histological examination revealed chronic cholecystitis with Rokitansky-Aschoff sinuses and fibromuscular tissue proliferation. There was also sporadic arteriolar occlusion associated with intimal thickening in the muscular layer of the gallbladder (Figure 4).

## 3. Discussion

Sorafenib is a molecular targeted agent that is already in widespread use worldwide for malignancies such as renal carcinoma, colon cancer, breast cancer, and HCC. There have been no reports to date of acalculous cholecystitis occurring during sorafenib therapy. However, six renal carcinoma cases and two HCC cases with complicating acute acalculous cholecystitis on Sorafenib had been reported to Bayer in Japan on the market between April 2008 and June 2010 (not published). Seven of eight cases were serious. There may be an increased incidence of acute acalculous cholecystitis. There have already been two reports of acalculous cholecystitis in patients treated with sunitinib, which is an angiogenesis inhibitor like sorafenib [5, 6], suggesting the potential for such events to occur with sorafenib.

Critically ill patients frequently develop acute acalculous cholecystitis following trauma or major surgery. Such patients then often go on to develop gangrene and perforation and have a higher mortality rate than patients with calculous cholecystitis [7, 8]. Common risk factors for acute acalculous cholecystitis include surgery, severe trauma, burns, and parenteral nutrition [9], but malignant metastases to the hepatic hilum [10], hepatic artery infusion of anticancer drugs [11], diabetes mellitus [12], and other conditions are also reportedly involved. However, in addition to having a good PS (0) when given sorafenib, the present patient did not have such risk factors or a history of concurrent diabetes mellitus, hypertension, ischemic heart disease, or thromboembolism. She also fully satisfied the eligibility criteria for sorafenib treatment, with good hepatic function (Child-Pugh Class A) and no marked cytopenia. We therefore strongly suspected that sorafenib was involved in this patient's acute acalculous cholecystitis.

Sorafenib presents a high risk for vascular adverse events such as hypertension, hemorrhage, and arterial thromboembolic events (ATEs), such as myocardial ischemia and myocardial infarction, mediated by the drug's inhibition of vascular endothelial growth factor (VEGF) [13]. The mechanism by which ATEs occurs is not yet understood, but it has been postulated that homeostasis between the vascular endothelium and platelets is disturbed by sorafenib-mediated damage to vascular endothelial cells, allowing platelets to aggregate more readily on the surface of the vascular endothelium [14].

Thrombi could not be confirmed in arterioles of the resected gallbladder because 45 days had passed since the onset of acute cholecystitis in this patient, but arteriolar occlusion associated with vascular endothelial thickening was seen sporadically. This type of vascular remodeling associated with arteriolar intimal thickening is induced by hypoxia [15], suggesting that the cause of the acute cholecystitis in this patient was gallbladder ischemia rather than calculus impaction. Based on the above, we postulated that acute acalculous cholecystitis occurred by the following mechanism in this patient. The vascular endothelium was damaged by administering sorafenib, and thrombi formed in the arterioles of the gallbladder. This resulted in a further reduction in gallbladder blood flow that was already reduced by chronic cholecystitis and caused acute acalculous cholecystitis.

Hepatocellular carcinoma often occurs in cirrhosis. Chronic cholecystitis and cholecystolithiasis are frequent complications of cirrhosis, and if acute cholecystitis occurs, it can easily become serious. We are concerned that there may be an increased incidence of acute cholecystitis with the expanding use of sorafenib and recommend caution when using this drug.
